# Predictors of 2-Year Post-Discharge Mortality in Hospitalized Older Patients

**DOI:** 10.3390/jcm13051352

**Published:** 2024-02-27

**Authors:** Christian Werner, Melanie Sturm, Patrick Heldmann, Tim Fleiner, Jürgen M. Bauer, Klaus Hauer

**Affiliations:** 1 Geriatric Centre, Heidelberg University Hospital, Agaplesion Bethanien Hospital Heidelberg, Rohrbacher Str. 149, 69216 Heidelberg, Germany; 2 Medical Faculty Heidelberg, Heidelberg University, Im Neuenheimer Feld 672, 69120 Heidelberg, Germany; 3 Network Aging Research (NAR), Heidelberg University, Bergheimer Str. 20, 69115 Heidelberg, Germany; 4 Division of Physiotherapy, Department of Applied Health Sciences, Hochschule für Gesundheit (University of Applied Sciences), Gesundheitscampus 6-8, 44801 Bochum, Germany; 5 Institute for Geriatric Research, Ulm University Medical Centre, Zollernring 26, 89073 Ulm, Germany; 6 Department of Geriatric Psychiatry and Psychotherapy, LVR-Hospital Cologne, Wilhelm-Griesinger Straße 23, 51109 Cologne, Germany; 7 Department of Clinical Gerontology, Robert-Bosch-Hospital, Auerbachstraße 110, 70376 Stuttgart, Germany

**Keywords:** mortality, hospitalization, risk factors, geriatrics, mobility

## Abstract

Background: Understanding prognostic factors for adverse health outcomes is clinically relevant for improving treatment decision-making processes, potentially leading to enhanced patient prognosis. This secondary analysis of a prospective observational study aimed to identify independent factors associated with 2-year post-discharge mortality in acutely hospitalized older patients. Methods: All-cause mortality and date of death of 115 patients (83.3 ± 6.3 years, females: *n* = 75, 65.2%) admitted to acute geriatric wards were determined two years after hospital discharge through telephone interviews. Potential prognostic factors measured at hospital admission included demographic and clinical characteristics, nutritional, cognitive, and psychological status, Fried frailty phenotype, functioning in activities of daily living, locomotor capacity, and 24 h in-hospital mobility and objectively measured physical activity (PA) behaviors. Results: The 2-year mortality rate was 36.7% (*n* = 41). Univariate and multivariate Cox proportional hazards regression models revealed that mean daily PA level (hazards ratio (HR) = 0.59, 95% confidence interval (CI) 0.90–1.00; *p* = 0.042), frailty (HR = 3.39, 95% CI 1.20–9.51; *p* = 0.020), and underweight, in contrast to overweight (HR = 3.10, 95% CI 1.07–9.01; *p* = 0.038), at hospital admission were independently predictive of post-discharge mortality. Conclusion: PA, frailty, and underweight at hospital admission should be considered when evaluating long-term survival prognosis, establishing risk profiles, and developing personalized care pathways in acute hospital care of older adults.

## 1. Introduction

The likelihood of hospitalization increases with age. Faced with the demographic changes of aging societies, healthcare systems are confronted with a growing number of older adults requiring acute care services. In some European countries, more than 40% of patients discharged from inpatient hospital care are aged 65 and older—about twice their proportion in the population [1].

Acutely hospitalized older adults are at high risk for functional decline [2], disability [3], institutionalization [4], and mortality after discharge [5]. Understanding prognostic factors for these adverse outcomes is clinically highly relevant. It facilitates the identification of those patients at high risk for poor outcomes, the efficient allocation of healthcare resources, and the development and implementation of tailored, patient-centered and effective treatment plans that can ultimately result in an improved prognosis for hospitalized older patients [6].

Previous studies have repeatedly identified older age [7,8], male gender [7,8], multimorbidity [7,8,9], polypharmacy [7], poor nutritional status [8,10,11,12], cognitive impairment [7,13], depressive symptoms [8,13], frailty [9,14], and poor functional status or locomotor capacity [7,14,15] as independent risk factors for all-cause mortality in older patients after hospital stay. However, research into the significance of patients’ mobility-related behavior during hospital stay as a potentially treatment-modifiable risk factor for post-discharge mortality is scarce. To our knowledge, only two studies have investigated this relationship, with both identifying lower mobility as risk factors for mortality after hospital discharge [16,17]. However, these studies have some limitations, in that mobility was either evaluated solely via nurse observations [16], which can be prone to proxy bias, or, when measured objectively via physical activity (PA) monitoring, the prediction model was adjusted only for a small number of patient characteristics (discharge diagnosis, comorbid conditions) [17]. In addition, there is an overall lack of studies that have investigated different constructs of real-world mobility measures (e.g., PA vs. life-space mobility) collected during hospitalization as predictors of post-discharge mortality in this patient population. 

The aim of this study was to identify independent factors associated with 2-year post-discharge mortality in hospitalized older patients collected at hospital admission, with a special focus on their actual in-hospital mobility behavior. We hypothesized that lower-mobility behavior would independently predict patients’ mortality risk after hospital discharge.

## 2. Materials and Methods

### 2.1. Study Design, Setting, and Population

This is a prospective secondary analysis of the PAGER study (“Physical Activity in Geriatric patients during Early Rehabilitation”) [18], which investigated mobility outcomes in hospitalized older patients receiving acute geriatric care (AGC). All patients admitted to the acute geriatrics wards of a German geriatric hospital from January to August 2019 were screened for eligibility. Inclusion criteria of the PAGER study were receipt of AGC (so-called “early rehabilitative geriatric complex treatment”) according to German Operation and Procedure Classification System, age ≥ 65 years, ability to walk 4 m with or without walking aid, sufficient German language skills, and written informed consent within 72 h after hospital admission. Exclusion criteria included severe cognitive impairment (Mini-Mental State Examination (MMSE) < 10 pt.) [19], delirium, terminal illness, and severe neurologic, cardiovascular, metabolic or psychiatric disorders compromising the ability to complete study procedures, and isolation for infection control. Details on the “early rehabilitative geriatric complex treatment” has been described elsewhere [18,19]. 

### 2.2. Outcome

All-cause mortality and date of death were determined two years after hospital discharge through telephone interviews conducted by a trained assessor. Mortality status was recorded as “alive” if a patient answered the call personally. When unable to reach the patient, the contact person (e.g., relative, legal representative, or caregiver) registered in the patient’s hospital record was contacted. These persons were asked whether the patient was still alive or had deceased, and if so, on what date. If neither the patient nor a contact person could be reached by telephone (three contact attempts each on different days) or the contact person did not consent to provide information about the patient’s status, the patient was considered as “lost to follow-up”. Survival time was calculated as time from hospital discharge to death. For patients still alive at the end of follow-up, survival time was censored at 2 years (730 days). 

### 2.3. Descriptive and Predictor Variables

Data were collected at hospital admission. Demographic characteristics, primary (admission reason) and secondary (comorbidities) diagnoses, and medications were derived from hospital records. Standardized patient interviews and testing procedures were used to assess nutritional status (Body Mass Index, BMI), cognitive status (MMSE) [19], depressive symptoms (Geriatric Depression Scale, 15-item version, GDS-15) [20], fear of falling (FoF, Short Falls Efficacy Scale-International, Short FES-I) [21], functioning in activities of daily living (ADL, Barthel Index) [22], and locomotor capacity (Short Physical Performance Battery, SPPB) [23]. Frailty was defined as meeting ≥ 3 criteria of the Fried frailty phenotype: weight loss (>4.5 kg in the past year), exhaustion (2 items from the Centre for Epidemiological Survey-Depression Scale), low PA (short version of the Minnesota Leisure Time Physical Activity Questionnaire: female < 270 kcal/week, male < 383 kcal/week), slowness (gender- and height-adjusted slow gait speed), and weakness (gender- and BMI-adjusted low handgrip strength) [24].

PA was measured using an inertial measurement unit (uSense activity monitor) [25,26] attached to the patient’s lower back for 48 h with waterproof adhesive foil within the first 72 h of AGC initiation (median 3.3, interquartile range (IQR) 0.5–23.8 h). The durations (min) of four activity categories (active, sedentary, walking, lying), step count, mean walking bout duration (s), and mean daily PA level (metabolic equivalent of tasks, METs (kcal/kg/h)) were extracted from the data processing software for the initial 24 h recordings to determine patients’ early in-hospital PA. Details on the data processing of the PA parameters have been described elsewhere [18,25,26].

In-hospital mobility was assessed by the interview-based Life-Space Assessment in Institutionalized Settings (LSA-IS). The LSA-IS documents the spatial extent of mobility across five distinct areas (1 = own room, 2 = within the ward, 3 = within the facility, 4 = immediate outdoor area of the facility, 5 = beyond the area of the facility) and the frequency of mobility within each area (1×/day, 2–3×/day, 4–5×/day, >5×/day) over the last day, also considering the degree of independence in mobility (personal support, equipment, without any support) [27].

### 2.4. Statistical Analysis

Data were analyzed using IBM SPSS Statistics for Windows, Version 29.0 (IBM Corp., Armonk, NY, USA). Crude mortality rate was calculated by dividing the number of deaths by the cumulative survival time and expressed as the number of deaths per 1000 person-years. Predictors of mortality were analyzed using univariate and multivariate Cox proportional hazards regression models. Covariates included age, sex, nutritional status (underweight—BMI < 23 kg/m^2^, normal—BMI = 23–30 kg/m^2^, overweight—BMI > 30 kg/m^2^) [28], comorbidities, medications, cognitive impairment (MMSE < 24 pt.), depressive symptoms (GDS-15 > 5 pt.) [29], FoF (low—Short FES-I = 7–8 pt., moderate—Short FES-I = 9–14 pt., high—Short FES-I ≥ 14 pt.) [30], ADL functioning (Barthel Index), frailty, locomotor capacity (SPPB), PA (activity duration (active + walking duration), mean daily PA level, step count, mean walking bout duration), and in-hospital mobility (LSA-IS). Those with a *p*-value of <0.10 in the univariate analysis were entered into a multivariate model. Multicollinearity within the model was checked by calculating tolerance and variance inflation factors (VIF), with tolerance < 0.1 and VIF > 10 indicative of multicollinearity. Hazard ratios (HRs) were given with 95% confidence intervals (95% CI), and covariates with a *p*-value of <0.05 were considered significant in the multivariate model. Multiple imputation by chained equations with predictive mean matching was used to impute missing data for covariates (20 imputations, 10 iterations), assuming that data were missing at random. This assumption was checked using a Little’s missing completely at random test (*p* = 0.450) and testing for differences in baseline characteristics, survival time and mortality status between patients with and without a complete data set (*p* = 0.055–0.947). The imputation model included all covariates, and the outcome and survival time were used as explanatory variables. Rubin’s rules were used to pool results over the imputed datasets.

## 3. Results

Out of the 155 patients enrolled in the primary PAGER study, the predictor variables and 24-month follow-up data on mortality status were obtained for 115 patients (no assessment at hospital admission—*n* = 16, deceased during hospital stay—*n* = 2, lost to follow-up—*n* = 22). Details on the flow of patient screening, recruitment, and enrolment of the PAGER participants have been published previously [18]. Detailed characteristics of the 115 patients (age = 83.3 ± 6.3 years; females: *n* = 75, 65.2%) included in this secondary analysis are provided in Table 1. 

During the 2-year follow-up, 41 (36.7%) persons died, representing a mortality rate of 277 (95% CI 198.8–375.8) deaths per 1000 person-years. Median survival time was 322 (IQR 104–560.5) days in deceased persons. Univariate analyses showed that nutritional status, comorbidities, cognitive and frailty status, ADL functioning, and mean daily PA level were associated (*p* < 0.10) with mortality (Table 2). In the multivariate analysis, a higher mean daily PA level (HR = 0.59, 95% CI 0.39–0.98; *p* = 0.042) was identified as an independent predictor of a lower 2-year post-discharge mortality risk, while frailty (HR = 3.39, 95% CI 1.21–9.51; *p* = 0.020) and underweight, in contrast to overweight (HR = 3.10, 95% CI 1.07–9.01; *p* = 0.038), were independently associated with an increased mortality risk. No multicollinearity was found between the variables in the multivariate model (tolerance = 0.481–0.937; VIF = 1.067–2.079).

## 4. Discussion

This study aimed at identifying predictors of the 2-year post-discharge mortality in hospitalized older adults collected in the early phase of hospital stay. Our results reveal that a lower mean daily PA level, frailty, and underweight were independent risk factors for all-cause mortality within 2 years after discharge.

The mortality rate observed in this study was 36.7%, which is similar to previous studies that found 2-year post-discharge mortality rates of approximately 36 to 43% for older adults admitted to acute geriatric hospital wards [31,32,33,34].

Lower objectively measured PA at hospital admission was an independent predictor of mortality after discharge. Each 0.1-MET decrement was associated with a roughly 40% higher 2-year mortality risk. To our knowledge, there is only one study that has linked the objectively measured PA behavior of hospitalized older patients with post-discharge mortality, which also revealed that lower PA at admission, as measured by the number of steps, was independently associated with an increased risk of 2-year mortality [17]. In hospitalized older adults, even short periods of low PA have been reported to rapidly induce significant physiological deconditioning [35], which in turn has been associated with an increased mortality risk following hospitalization [36,37], providing a plausible explanation for the observed relationship between the lower 24 h PA at hospital admission and the post-discharge mortality risk. Our findings suggest that early PA promotion should be a focus in acute hospital care for older patients, as it can make an important contribution to long-term survival in this patient population. 

No significant association between patients’ spatial extent, frequency, and/or independence of mobility (LSA-IS) and mortality were observed. This is in contrast to previous research; however, this previous work did not consider objectively measured PA as another construct of real-world mobility behavior in its analysis as a potential risk factor [16]. Our results suggest that PA, defined as any bodily movement in daily life that requires energy expenditure, and which was objectively measured in this study, has a higher prognostic value for post-discharge mortality than patients’ self-reported in-hospital mobility behavior. 

Frailty has previously been identified as a risk factor for long-term mortality in older patients after hospital discharge [9,14]. The present study confirms this association by revealing that frail patients faced a 3.4-fold higher mortality risk compared to non-frail patients. This finding supports the importance of integrating routine frailty assessments into the hospital care of older patients to allow for more precise patient risk profiling and to support the development of personalized care pathways tailored to the needs of frail patients.

Patients with an underweight BMI showed a 3.1-fold higher mortality risk in comparison to those with an overweight BMI. This is consistent with previous research, which has shown a higher post-discharge mortality risk associated with underweight in comparison to overweight [12], and with lower BMIs in general among hospitalized older adults [10,11]. This also supports the “obesity paradox”, whereby older overweight individuals may paradoxically experience better outcomes than their normal-weight or underweight counterparts [38]. These findings highlight the importance of placing a special focus on nutrition in hospitalized older patients to prevent weight loss during the hospital stay, and that the BMI can provide clinicians with valuable information for developing tailored, patient-centered treatment approaches.

The present study has some limitations. First, the sample size of this secondary analysis of the PAGER study, which was not specifically designed to evaluate predictors of participants’ 2-year post-discharge mortality, was rather small, limiting the statistical power and the generalizability of the results. Second, the study was conducted as a single-center trial conducted in AGC wards of a German geriatric hospital, thus the results may not be generalizable to older patients treated in other hospital wards due to contextual and regional differences in healthcare. Third, patients with severe cognitive and/or gait impairments were excluded, and the results may not be generalizable to these most severely impaired individuals, who may be at the highest mortality risk. Fourth, cognitive impairment and depressive symptoms were assessed using screening tools (MMSE, GDS-15), and therefore may not reflect the actual (neuro-)psychological status of the patients. Fifth, mortality status could not be obtained through telephone interview for 24 patients (16%). Although they did not significantly differ in any participant characteristics at baseline, a potential risk of bias could be caused by this exclusion. Finally, although the statistical model was controlled for several important confounders, residual confounding is still possible, and it cannot be ruled out that other unmeasured factors may have influenced the associations found (e.g., patient- vs. staff-initiated in-hospital mobility behavior; care process, health behaviors, and lifestyle changes after hospital discharge). 

## 5. Conclusions

This secondary analysis showed that lower PA, frailty, and underweight are important risk factors for all-cause mortality in hospitalized older patients two years post-discharge. These factors should be proactively assessed and used to derive tailored, patient-centered therapeutic strategies and care pathways that may improve the long-term survival of older patients after hospitalization.

## Figures and Tables

**Table 1:** Participant characteristics.

Variable	*n* = 115 ^1^
Age, years	83.3 ± 6.3
Female, *n*	75 (65.2)
Living situation before admission, *n*	
Community-dwelling	102 (88.7)
Assisted living	11 (9.6)
Nursing home	2 (1.7)
Primary diagnosis for admission, *n*	
Musculoskeletal	31 (27.0)
Neurological	20 (17.4)
Infectious	16 (13.9)
Cardiovascular	11 (9.6)
Gastrointestinal	9 (7.8)
General health deterioration	9 (7.8)
Neuromusculoskeletal	4 (3.5)
Others	15 (13.0)
Comorbidities, *n*	10.2 ± 5.3
Medications, *n*	10.4 ± 4.1
BMI, kg/m^2^ (*n* = 105)	26.3 ± 5.1
Nutritional status, *n*	
Underweight (BMI < 23 kg/m^2^)	29 (25.2)
Normal (BMI 23–30 kg/m^2^)	55 (47.8)
Overweight (BMI > 30 kg/m^2^)	21 (18.3)
MMSE, pt.	22.4 ± 4.8
Cognitive impairment (MMSE < 24 pt.), *n*	61 (53.0)
GDS-15, pt.	4.8 ± 3.2
Depressive symptoms (GDS-15 > 5 pt.), *n*	40 (34.8)
Short FES-I, pt. (*n* = 107)	12 [8–17]
Low FoF (Short FES-I = 7–8 pt.), *n*	36 (31.1)
Moderate FoF (Short FES-I = 9–14 pt.), *n*	25 (21.7)
High FoF (Short FES-I = 14–28 pt.), *n*	46 (40.0)
Barthel Index, pt.	55 [45–72.5]
Fried frailty phenotype, *n* (*n* = 100)	69 (69.0)
SPPB, pt. *(n* = 105)	3.9 ± 2.3
Physical activity (*n* = 112)	
Active, min	30.1 [15.9–53.0]
Sedentary, min	607.7 [454.2–711.0]
Walking, min	5.5 [1.0–16.3]
Lying, min	777.8 [665.7–936.7]
Step count, *n*	499 [73–1352]
Mean walking bout duration, s	7.6 [4.0–11.3]
LSA-IS, pt. (*n* = 113)	12.6 ± 8.3

BMI, Body Mass Index; MMSE, Mini-Mental State Examination; GDS-15, Geriatric Depression Scale, 15-item version; Short FES-I, Short Falls Efficacy Scale-International; FoF, fear of falling; SPPB, Short Physical Performance Battery; LSA-IS, Life-Space Assessment in Institutionalized Settings. Data given as *n* (%), median [interquartile range], or mean ± standard deviation. ^1^ *n* = 115, unless otherwise indicated.

**Table 2:** Univariate and multivariate Cox proportional hazards regression models for 2-year post-discharge mortality.

Characteristics	Alive (*n* = 74)	Deceased (*n* = 41)	Univariate Analysis		Multivariate Analysis	
			HR (95% CI)	*p*	HR (95% CI)	*p*
Age, years	82.9 ± 6.4	84.1 ± 6.0	1.03 (0.98–1.08)	0.323		
Female, *n*	50 (67.6)	25 (61.0)	0.81 (0.43–1.52)	0.520		
Nutritional status, *n*						
Overweight (ref.)	18 (24.3)	6 (14.6)	-	-	-	-
Normal	41 (55.4)	19 (46.3)	1.34 (0.52–3.46)	0.546	1.74 (0.64–4.78)	0.279
Underweight	15 (20.3)	16 (39.0)	2.37 (0.92–6.26)	0.075	3.10 (1.07–9.01)	0.038
Comorbidities, *n*	9.6 ± 4.8	11.2 ± 6.1	1.05 (0.99–1.12)	0.077	1.05 (0.98–1.12)	0.139
Medications, *n*	10.2 ± 3.8	10.8 ± 4.7	1.03 (0.96–1.12)	0.399		
Cognitive impairment, *n*	34 (45.9)	27 (65.9)	1.87 (0.98–3.56)	0.058	1.41 (0.70–2.80)	0.335
Depressive symptoms, *n*	26 (35.1)	14 (34.1)	0.95 (0.50–1.81)	0.872		
Fear of falling, *n*						
Low (ref.)	23 (31.1)	15 (36.6)	-	-		
Moderate	18 (24.3)	9 (22.0)	0.88 (0.38–2.02)	0.756		
High	33 (44.6)	17 (41.5)	0.85 (0.42–1.74)	0.661		
Frailty, *n*	42 (56.8)	36 (87.8)	4.05 (1.60–10.22)	0.003	3.39 (1.21–9.51)	0.020
SPPB, pt.	4.1 ± 2.5	3.4 ± 2.2	0.89 (0.77–1.03)	0.119		
Barthel Index, pt.	52.6 ± 17.9	46.0 ± 16.1	0.98 (0.97–1.00)	0.066	1.01 (0.99–1.03)	0.562
Physical activity						
Activity duration, min	55.6 ± 44.1	42.8 ± 40.8	0.99 (0.99–1.00)	0.133		
Mean daily PA level, MET *	1.46 ± 0.08	1.42 ± 0.08	0.59 (0.39–0.90)	0.015	0.59 (0.36–0.98)	0.042
Step count, *n* ^†^	1199 ± 1628	1000 ± 1617	0.83 (0.58–1.19)	0.307		
Mean walking bout duration, *s*	8.5 ± 5.2	8.5 ± 5.7	0.86 (1.00–1.06)	0.919		
LSM-IS, pt.	13.1 ± 9.0	11.5 ± 6.9	0.98 (0.94–1.02)	0.353		

HR, hazard ratio; ref., reference category; SPPB, Short Physical Performance Battery; PA, physical activity; LSA-IS, Life-Space Assessment in Institutionalized Settings. Descriptive data are given as *n* (%) or mean ± standard deviation. * HR given for an increase of 0.1 METs. ^†^ Natural log-transformed for Cox regression models.

## Data Availability

The datasets used and/or analyzed in this secondary analysis are available from the corresponding author on reasonable request.
